# Short term risk of non-fatal and fatal suicidal behaviours: the predictive validity of the Columbia-Suicide Severity Rating Scale in a Swedish adult psychiatric population with a recent episode of self-harm

**DOI:** 10.1186/s12888-018-1883-8

**Published:** 2018-10-01

**Authors:** Åsa U. Lindh, Margda Waern, Karin Beckman, Ellinor Salander Renberg, Marie Dahlin, Bo Runeson

**Affiliations:** 1 Centre for Psychiatry Research, Department of Clinical Neuroscience, Karolinska Institutet, & Stockholm Health Care Services, Stockholm County Council, S:t Görans Hospital, Vårdvägen 1, SE-112 81 Stockholm, Sweden; 2 Department of Psychiatry and Neurochemistry, University of Göteborg, Gothenburg, Sweden; 3 Department of Clinical Sciences, Division of Psychiatry, University of Umeå, Umeå, Sweden

**Keywords:** Self-harm, Suicide, Risk factors, Classification, Outcome

## Abstract

**Background:**

The Columbia-Suicide Severity Rating Scale (C-SSRS) is a relatively new instrument for the assessment of suicidal ideation and behaviour that is widely used in clinical and research settings. The predictive properties of the C-SSRS have mainly been evaluated in young US populations. We wanted to examine the instrument’s predictive validity in a Swedish cohort of adults seeking psychiatric emergency services after an episode of self-harm.

**Methods:**

Prospective cohort study of patients (*n* = 804) presenting for psychiatric emergency assessment after an episode of self-harm with or without suicidal intent. Suicidal ideation and behaviours at baseline were rated with the C-SSRS and subsequent non-fatal and fatal suicide attempts within 6 months were identified by record review. Logistic regression was used to evaluate separate ideation items and total scores as predictors of non-fatal and fatal suicide attempts. Receiver operating characteristics (ROC) curves were constructed for the suicidal ideation (SI) intensity score and the C-SSRS total score.

**Results:**

In this cohort, the median age at baseline was 33 years, 67% were women and 68% had made at least one suicide attempt prior to the index attempt. At least one non-fatal or fatal suicide attempt was recorded during follow-up for 165 persons (20.5%). The single C-SSRS items frequency, duration and deterrents were associated with this composite outcome; controllability and reasons were not. In a logistic regression model adjusted for previous history of suicide attempt, SI intensity score was a significant predictor of a non-fatal or fatal suicide attempt (OR 1.08; 95% CI 1.03–1.12). ROC analysis showed that the SI intensity score was somewhat better than chance in correctly classifying the outcome (AUC 0.62, 95% CI 0.57–0.66). The corresponding figures for the C-SSRS total score were 0.65, 95% CI 0.60–0.69.

**Conclusions:**

The C-SSRS items frequency, duration and deterrents were associated with elevated short term risk in this adult psychiatric cohort, as were both the SI intensity score and the C-SSRS total score. However, the ability to correctly predict future suicidal behaviour was limited for both scores.

## Background

Suicide accounts for more than 1% of all deaths worldwide, making it the 15th leading cause of death. Non-fatal self-harm is more common, with an estimated annual prevalence of 4/1000 adults [1]. People who have self-harmed are at increased risk of future self-harm and of dying by suicide [2–8]. The one-year rate of repetition has been estimated at 16% for non-fatal attempts and 1.6–1.8% for fatal attempts [9, 10].

Given the suffering associated with suicidal behaviours, effort has been put into understanding and predicting the phenomenon, with the ultimate goal of prevention. Risk factors for suicide and suicide attempt have been identified in numerous cohorts, but clinically useful tools for prediction at the individual level have yet to be identified [11–13]. In a recent review of the diagnostic accuracy regarding suicide and suicide attempt for several suicide risk assessment instruments, the Columbia-Suicide Severity Rating Scale (C-SSRS) was highlighted as an instrument in need of testing in larger populations [14]. The C-SSRS is a relatively new instrument designed for classification and grading of suicidal ideation and behaviours [15, 16]. Suicidal ideation (SI) is assessed with regard to severity and intensity. Previous studies performed mainly in the US and in adolescent and young adult age groups suggest that both SI severity [15, 17–20], SI intensity [17, 20, 21] and the C-SSRS total score [19] are predictive of suicidal behaviours. The instrument has solid psychometric properties [15, 19, 22, 23].

We aimed to evaluate the predictive ability of the C-SSRS in a large adult sample of patients seeking or being referred to psychiatric emergency care after an episode of self-harm with or without suicide intent. We chose a follow-up time of six months as this is the period with the highest risk of repetition [7, 24] and a time span relevant for treatment planning [25–27]. Since the cohort had a large proportion of actual suicide attempts at the index episode, we assumed there would be relatively little variation in the ratings on severity of suicidal ideation compared to the intensity, which could make the SI intensity score or the separate intensity items more suited as predictors. To be of predictive use in a population similar to our sample, an item or risk scale must show predictive ability when suicide attempts are adjusted for, since this is already acknowledged as one of the most important risk factors for future suicide attempt and suicide [4, 8, 28, 29]. Therefore, we tested whether the SI intensity score, the separate intensity items and the single item *most severe ideation* would predict a repeated suicide attempt also after adjustment for previous attempts.

## Methods

### Participants

The present study uses data from a Swedish multi-centre study conducted at S:t Görans Hospital (Karolinska Institutet, Stockholm), Sahlgrenska University Hospital (Gothenburg) and Umea University Hospital (Umeå). Adult patients (aged 18 and above) presenting for or being referred to a psychiatric assessment after an episode of self-harm were considered for participation if they were able to take part in an interview. There were no exclusion criteria regarding specific diagnoses, but patients with symptoms interfering with verbal communication (e g severe psychotic symptoms, aggression, confusion, severe somatic conditions and severe cognitive impairment) were not considered for participation. To enable follow-up by medical records, participants were required to have a Swedish personal identity number and to be a registered resident of the catchment area for psychiatric services at one of the participating hospitals. Participants were interviewed by mental health staff (psychiatrists, psychologist and psychiatric nurses) specially trained in the application of the assessment instrument. All interviews took place daytime from April 25, 2012 to April 6, 2016, in most cases within a couple of days of the index episode.

### Scorings and outcome

#### Patient data

The following variables were collected during the interview: sex, age, current occupation (work/student/unemployed/on sick leave/receiving disability pension/retired), living alone/together with someone, current outpatient treatment for psychiatric disorder (including both primary care and specialized mental health care), psychiatric hospitalization during the past 3 months, previous suicide attempt (SA), previous non-suicidal injury (NSSI) and type of index episode (SA or NSSI). Suicide attempt was defined as a potentially self-injurious act committed with at least some wish to die as a result of the act [15, 30]. The questions from the behavioural part of the C-SSRS were used to assess intent. Non-suicidal self-injury was defined as a self-injurious act with no wish to die as a result [31].

#### Suicidal ideation and behaviour

We used the clinician-administered version of the C-SSRS (baseline/screening version) to assess suicidal ideation (SI) within the past 30 days, suicidal behaviour for the past three months and lifetime ratings for both ideation and behaviour. Suicidal ideation severity is assessed with five yes/no questions summarized in the single item *most severe ideation*, scored 0–5 where 0 corresponds to no suicidal ideation, 1 to a wish to die and 5 to active suicidal ideation with a specific plan and intent to act. The intensity of suicidal ideation is assessed if some degree of SI severity is endorsed. The SI intensity score was derived from the ratings for the following items: frequency, duration, controllability, deterrents and reasons for ideation. Participants denying any degree of suicidal ideation were given 0 points for each of these items. For the items *frequency* and *duration* (scored 1–5), high ratings correspond to high frequency and long duration. For the items *controllability* and *deterrents* (scored 0–5), high ratings indicate a low level of controllability, and little or no deterring effect of factors that could stop someone from acting on suicidal thoughts. In line with a previous study [21], participants stating that there was nothing deterring them from acting upon suicidal thoughts were given a rating of 5 on this item. For the item *reasons for ideation* (scored 0–5), low ratings indicate that a wish to affect others is the main reason for suicidal ideation whereas a high rating indicates that the motive is to end one’s own pain. Ratings for the intensity items were summed to yield a SI intensity score that could range from 0 to 25.

In the subscale assessing suicidal behaviour, the participants state whether or not they have engaged in actual, aborted or interrupted suicide attempts, NSSI or preparatory behaviour. The total number of episodes is recorded for each type of behaviour. For actual suicide attempts, actual lethality/medical damage is scored on a six-point ordinal scale. If actual lethality is rated 0, potential lethality is scored from 0 to 2.

The C-SSRS was constructed as an instrument for classification, and there are thus no instructions for calculating a total score. Regarding the ideation items, we used the above-described values. For the behaviour items, we chose to categorize the number of attempts in three groups: 0 (no attempts), 1 (1–2 attempts) and 2 (three or more attempts). If NSSI and/or preparatory behaviour were recorded, 1 point each was given. Regarding lethality of the attempt, the score for the most recent attempt was used. Using this method, the C-SSRS total score has a possible range of 0–42.

Regarding internal consistency, Cronbach’s α was 0.64 for the five initial questions assessing SI severity and 0.49 for the SI intensity score. Inter-rater reliability was assessed using weighted kappa for items with even distribution of responses. Prevalence-adjusted, bias-adjusted kappa (PABAK) was calculated when responses were unevenly distributed. The prevalence-adjusted, bias-adjusted kappa for the items *most severe ideation*, frequency, duration, controllability and reasons for ideation ranged from 0.82–0.95 whereas deterrents had a PABAK of 0.63. The PABAK for the behaviour items ranged from 0.70–0.90.

#### Outcome

The composite outcome was any non-fatal or fatal suicide attempt within six months of the index episode, as identified by medical record review. All available entries recorded during the follow-up period were examined. An act of self-injury that involved at least some wish to die was classified as a suicide attempt. Fatal attempts were defined as deaths that were specifically identified as suicides in the medical records. The electronic medical record systems used at all three sites are all linked to the Swedish population register. This register includes all Swedish residents and is updated daily, with almost 100% of deaths being registered within 30 days [32]. While the population register does not contain information about cause of death, it does identify persons who are still alive after 6 months, including those with no health care contact after the index episode.

#### Statistical analysis

Data analyses were conducted using the Statistical Package for the Social Sciences (SPSS) version 22.0. We used past month ratings for all analyses involving suicidal ideation and past three month ratings for those involving suicidal behaviour. Ratings on the single item *most severe suicidal ideation* as well as the SI intensity score and the separate intensity items (frequency, duration, controllability, deterrents and reasons) were entered in separate bivariate and multivariable logistic regression models, as single predictors of the composite outcome (non-fatal or fatal attempt) and adjusted for age, sex and suicide attempt prior to the index episode. The C-SSRS total score was analysed without adjustment for prior suicide attempts as this factor is incorporated in the total score. Those with missing values were excluded from the regression analyses.

Receiving operating characteristic analyses were performed for the SI intensity score as well as the C-SSRS total score to assess the area under the curve and identify potential cut-off scores.

## Results

### The cohort at baseline

Of 1138 eligible participants, 804 (71%) agreed to participate in the study. Baseline characteristics of the study cohort are shown in Table 1.

**Table 1:** Baseline characteristics of adults presenting at psychiatric emergency services in connection with an episode of self-harm in a Swedish multicentre study 2012–2016 (*N* = 804)

	Mean	SD
Age	38 years	18
	N	%
Women	541	67%
Current occupation		
Work/student	325	40%
Unemployed/ sick leave/disability pension	395	49%
Retired	84	10%
Living alone	424	53%
Current mental health treatment^a^	573	71%
Inpatient care past 3 months	231	29%
Previous suicide attempt	544	68%
Previous non-suicidal self-injury	421	53%
Suicide attempt at index	666	83%
Psychiatric hospitalization at index^b^	750	93%
Mood disorder at index (F30–39)^c^	295	37%
Substance use disorder at index (F10–19)^c^	172	21%

^a^: having an ongoing contact with primary or psychiatric care with treatment for a psychiatric condition

^b^: defined as at least one night’s admission to inpatient care

^c^: diagnosis in primary or secondary position

*SD* Standard Deviation

Two thirds of the participants were women and the age range was 18–95 years, with a median of 33 years (interquartile range 23–50). Almost half of the participants were either unemployed or had sickness benefits or disability pension, and almost three quarters were already in outpatient treatment for a psychiatric disorder. Two thirds of the participants had a history of at least one previous suicide attempt, and half of them had at least one previous episode of non-suicidal self-injury. Most participants were admitted to psychiatric hospital care after the index episode; median stay was 9 days (range 1–238 days). A clinical diagnosis of mood disorder was recorded in one third of the participants, and one fifth had a substance use disorder.

Table 2 shows the baseline ratings on the C-SSRS. A majority of the participants (*n* = 689, 86%) reported high ratings (4–5) on the C-SSRS item *most severe ideation* during the 30 days before the index episode with a mean score of 4.5 (SD 1.2) for the whole group. Corresponding figures were lower for all intensity items except *reasons for ideation*. Lifetime ratings were very similar to those reported for the past 30 days. Twenty-nine participants, twelve of which had made a suicide attempt at index, denied any suicidal ideation during the past month. Five or more actual suicide attempts (range 5–50) were recorded during the past three months for 19 participants. Six of these stated that they had made many hundreds of actual, aborted and interrupted attempts during their lifetime.

**Table 2:** Baseline C-SSRS ratings in adults presenting at psychiatric emergency services in connection with an episode of self-harm in a Swedish multicentre study 2012–2016 (N = 804)

C-SSRS ratings, past 30 days	Mean	SD
Most severe ideation, range 0–5	4.5	1.2
Intensity items, range 0–5		
Frequency	3.5	1.6
Duration	2.9	1.5
Controllability	3.2	1.7
Deterrents	3.3	1.7
Reasons	4.4	1.3
SI intensity score, range 0–25	17.2	5.3
C-SSRS total score^a^, range 0–42	26	7
C-SSRS ratings, lifetime	Mean	SD
Most severe ideation, range 0–5	4.7	0.9
Intensity items, range 0–5		
Frequency	3.9	1.4
Duration	3.3	3.3
Controllability	3.4	3.4
Deterrents	3.5	3.5
Reasons	4.6	0.9
SI intensity score, range 0–25	18.6	4.8
C-SSRS total score, range 0–42	29	7

*C-SSRS* Columbia-Suicide Severity Rating Scale, *SD* Standard deviation, *SI* Suicidal ideation

^a^C-SSRS behavioural items are rated for past three months

### Non-fatal and fatal suicide attempts during follow-up

During the six months follow-up, 165 (20.5%) participants made a non-fatal or fatal suicide attempt. Of these, 159 persons (19.8% of the cohort; 18% of all men, 20.5% of all women) made at least one non-fatal suicide attempt and ten persons (1.2% of the cohort; 1.9% of all men, 0.9% of all women) died by suicide. Four persons made a suicide attempt and survived, but died by suicide later in the follow-up period. Nine persons died from causes other than suicide. No entries appeared in the medical records after the index attempt in 15 persons (1.9% of the total sample), which in most cases was due to the participant moving from the catchment area. All 15 were still alive at the end of the follow-up period.

### Associations between C-SSRS ratings and future suicide attempts

The single item *most severe ideation* was significantly associated with a non-fatal or fatal suicide attempt during the six month follow-up, but this did not withstand adjustment for age, sex and suicide attempt prior to the index episode (Table 3).

**Table 3:** C-SSRS items, intensity score and total score as predictors of non-fatal and fatal suicide attempt during six month follow-up among adult patients presenting at psychiatric emergency services in connection with an episode of self-harm in a Swedish multicentre study 2012–2016

Item	N with score (n with outcome)	Actual attempts, non-fatal and fatal, during 6 month follow-up; unadjusted				Actual attempts, non-fatal and fatal, during 6 month follow-up; adjusted models^a^			
		OR (95% CI)	*p*	C&S R^2^	N’s R^2^	OR (95% CI)	*p*	C&S R^2^	N’s R^2^
Most severe ideation	801(165)	1.2 (1.001–1.4)	0.048	0.006	0.009	1.2 (0.9–1.4)	0.06	0.051	0.079
SI Intensity score	800 (165)	1.1 (1.04–1.1)	< 0.001	0.020	0.034	1.07 (1.03–1.1)	0.001	0.059	0.093
SI Frequency	798 (165)	1.3 (1.1–1.4)	< 0.001	0.020	0.031	1.2 (1.1–1.4)	0.002	0.057	0.089
SI Duration	798 (165)	1.2 (1.1–1.4)	< 0.001	0.017	0.026	1.2 (1.03–1.3)	0.01	0.053	0.083
SI Controllability	796 (164)	1.2 (1.1–1.3)	0.002	0.013	0.020	1.1 (1.01–1.3)	0.03	0.052	0.081
SI Deterrents	796 (164)	1.1 (0.9–1.2)	0.05	0.005	0.008	1.1 (1.03–1.3)	0.02	0.054	0.084
SI Reasons	794 (162)	1.1 (0.9–1.3)	0.2	0.002	0.003	1.1 (0.9–1.3)	0.3	0.046	0.073
C-SSRS total score	802 (165)	1.08 (1.05–1.1)	< 0.001	0.033	0.052	1.08 (1.04–1.1)	< 0.001	0.049	0.077

^a^Adjusted for age, sex, suicide attempt prior to index episode; C-SSRS total score adjusted for age and sex only

*C-SSRS* Columbia-Suicide Severity Rating Scale, *SI* Suicidal ideation, *OR* Odds ratio, *CI* Confidence interval, *C&S* Cox & Snell, *N’s* Nagelkerke’s

**Fig. 1:**
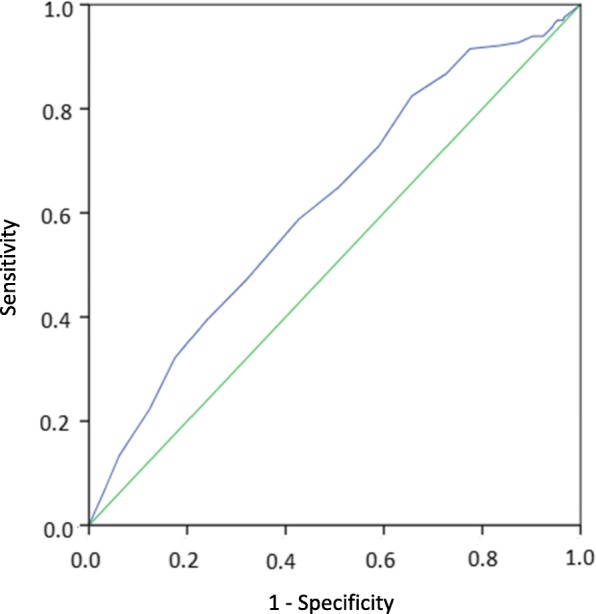
SI intensity score as predictor of a non-fatal or fatal attempt within 6 months in adult patients presenting at psychiatric emergency services in connection with an episode of self-harm in a Swedish multicentre study 2012–2016. AUC: 0.62 (95% CI 0.57–0.67), *p* < 0.001

**Fig. 2:**
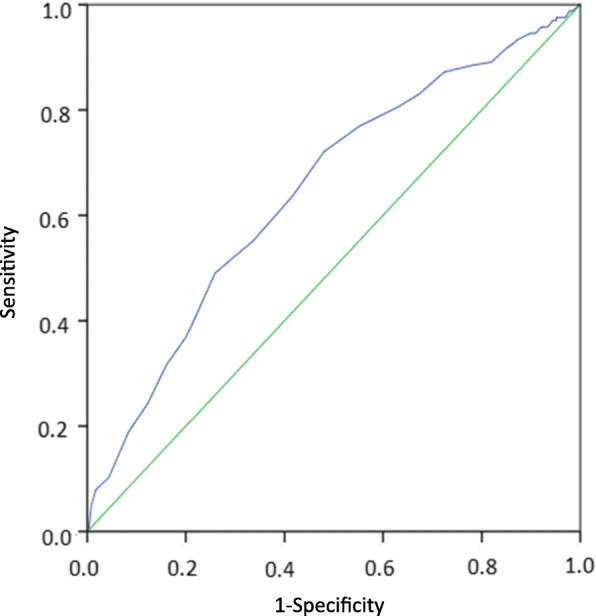
C-SSRS total score recent time as predictor of a non-fatal or fatal attempt within 6 months in adult patients presenting at psychiatric emergency services in connection with an episode of self-harm in a Swedish multicentre study 2012–2016. AUC 0.65 (95% CI 0.60–0.69), p < 0.001

The separate intensity items frequency, duration and controllability were significant predictors of a future attempt in unadjusted analyses. After adjustment for sex, age and prior suicide attempt all intensity items except reasons for ideation were significantly associated with the outcome with the odds increasing by 20% for each one-step increment in frequency and duration, and by 10% for each one-step increment in controllability and deterrents. The SI intensity score and the C-SSRS total score were also significantly associated with a future attempt before and after adjustment, with the odds increasing with 7 and 8% for each one-step increment, respectively. Figure 1 shows the ROC curve for the SI intensity score as a predictor of non-fatal or fatal suicide attempt within six months. The area under the curve (AUC) was 0.62 (95% CI 0.57–0.67), *p* < 0.001). A cut-off of 18.5 gave a sensitivity of 59% and a specificity of 57% in predicting a non-fatal or fatal suicide attempt. For the C-SSRS total score (Fig. 2), the AUC was 0.65 (95% CI 0.60–0.69, p < 0.001) and a cut-off of 28.5 gave a sensitivity of 69% and a specificity of 54%. Table 4 shows sensitivity and specificity over a range of cut-off values for the SI intensity score. Corresponding values for the C-SSRS total score are shown in Table 5.

**Table 4:** Sensitivity and specificity at different cut-off levels for baseline C-SSRS ratings on SI intensity score in predicting non-fatal and fatal suicide attempt during six month follow-up among adult patients presenting at psychiatric emergency services in connection with an episode of self-harm in a Swedish multicentre study 2012–2016

C-SSRS Intensity score, cut-off	Sensitivity	Specificity
15.5	82	34
16.6	73	41
17.5	65	49
18.5	59	57
19.5	47	68

*C-SSRS* Columbia-Suicide Severity Rating Scale, *SI* Suicidal ideation

**Table 5:** Sensitivity and specificity at different cut-off levels for baseline C-SSRS total score in predicting non-fatal and fatal suicide attempt during six month follow-up among adult patients presenting at psychiatric emergency services in connection with an episode of self-harm in a Swedish multicentre study 2012–2016

C-SSRS total score, cut-off	Sensitivity	Specificity
25.5	77	45
26.5	72	52
27.5	64	58
28.5	55	66
29.5	49	74

*C-SSRS* Columbia-Suicide Severity Rating Scale

## Discussion

One fifth of the participants in this large adult cohort made a non-fatal or fatal suicide attempt during the six-month follow-up. The C-SSRS total score and the SI intensity score were significant predictors of suicide attempt within six months, as were the single intensity items frequency, duration and deterrents after taking prior history of attempt into consideration. The overall ability of the SI intensity score to correctly distinguish between those who would and would not make non-fatal or fatal attempts within six months was only somewhat better than chance and the C-SSRS total score performed in a similar fashion.

The ratings on the item *most severe ideation* were high and uniform in this cohort and this item was not a significant predictor of suicide attempt within six months after adjustment for age, sex and prior attempt. This contrasts with results from some previous studies on the C-SSRS. In a treatment study of 124 teenagers who had made an actual or interrupted suicide attempt during the 90 days before inclusion, the most severe suicidal ideation during lifetime (which may or may not coincide with the index episode) predicted suicide attempt during six months follow-up [15]. In a retrospective medical record review of 473 consecutive psychiatric emergency cases with a follow-up time of 18 months, *most severe ideation* was predictive of suicide attempt over and above the impact of a previous suicide attempt [17]. Proportions with suicide attempts during follow-up were lower in these two cohorts (13% and 8%, respectively), suggesting a broader range of psychopathology. Under such circumstances, severe suicidal ideation could constitute a significant marker of risk. This would not be the case, however, in a cohort like ours with a high prevalence of severe ideation.

Regarding SI intensity, our finding that frequency, duration and deterrents were predictive of a non-fatal or fatal suicide attempt is in line with previous studies. In the above-cited retrospective study [17], the SI intensity score and the intensity item frequency were predictive of suicide attempt during the 18 month follow-up. In a cohort of 178 consecutive cases of adolescents seeking psychiatric emergency services, the SI intensity score and the intensity item duration were predictive of suicide attempt during 12 month follow-up [21]. Both frequency and duration of ideation have been suggested as markers of a ruminative process [17, 21]. Rumination has been correlated with both suicidal ideation [33] and suicide attempts [34]; the latter however not shown in prospective studies [35]. In a Danish study of 85 teenagers with suicidal behaviour or severe suicide thoughts, the single intensity item deterrents was associated with an actual suicide attempt with an almost three-fold increase in odds during a mean follow-up time of 80 days, when adjusting for actual attempts at baseline [20].

Even though we could demonstrate significant associations between different aspects of the C-SSRS and the outcome, in comparison with previous prediction studies our point estimates for all odds ratios are low, with narrow confidence intervals. In no instance did the upper limit of the 95% confidence interval exceed 1.4. Consequently, there is a low probability of a true population odds ratio greatly exceeding 1. It is unlikely that the factors studied are of major importance for an accurate clinical risk assessment after self-harm. This is also signalled by the fact that the regression models explain a very small proportion of the variance in the outcome.

The ROC curve for the C-SSRS total score as predictor had an AUC of 0.65 in the present study as compared with 0.76 in an adult psychiatric inpatient population [19]. While the same observation period was employed in both studies, comparison is difficult since the outcome of the latter study included also less serious behaviours (aborted and interrupted attempts and preparatory behaviour). Further, the proportion of actual suicide attempts was not presented in that study, nor was the proportion of patients with previous self-harm.

The Cronbach’s α for the SI intensity scale was 0.49, indicating limited internal consistency. This finding is similar to that of Youngstrom et al. [22] and might indicate that the SI intensity scale measures more than one underlying construct. These findings, however, contrast with the higher α values (0.73–0.95) presented by Posner and co-workers [15]. The interrater reliability for the different items varied from excellent for the items *most severe ideation*, frequency and duration to moderate/substantial for the item deterrents. This could indicate that the question about deterrents is more complex and that the others are more straightforward and easy to understand for both the patient and the interviewer.

In recent years, several authors have highlighted some problematic aspects of suicide risk assessment in general, e g that suicide risk is largely influenced by factors that are not yet present at the time of assessment [36]. Further, the categorization of patients in high- or low-risk groups is problematic since the incidence of suicide in the high-risk groups, as they have been defined, still is too low to motivate highly interfering interventions (e g involuntary treatment) and the low-risk group is large, meaning that many who die by suicide will have been classified as low-risk [11, 36]. In a recent systematic review of risk scales, even instruments with high sensitivity tended to have a low positive predictive value and no single instrument could be recommended for routine clinical work [37]. Another issue in the field of suicide research has been the lack of uniform terminology [30, 38, 39]. The C-SSRS makes an important contribution to the clarification of suicide-related behaviours. However, it has been argued that the C-SSRS does not cover all possible combinations of ideation and behaviour, and that the questions and instructions could be misinterpreted [40]. For example, a few participants in our study acknowledged some degree of suicidal intention in connection with an attempt that occurred within the past few days, yet negated any suicidal ideation during the past month.

### Strengths

This is the first study prospectively evaluating the predictive performance of the C-SSRS in an adult psychiatric population outside the US, contributing to the evidence base for this widely used instrument. The size of the cohort and the small proportion of participants lacking follow-up data are major strengths. The narrow confidence intervals indicate that non-significant results are not likely to be due to insufficient statistical power. The generous inclusion criteria, few exclusion criteria and high participation rate imply adequate external validity. Further, the age range is wide rendering results more relevant to clinical situations involving adult emergency psychiatric populations. The follow-up time of six months is shorter, and thus more clinically relevant, than in most prediction studies published to date [11, 14, 26]. Experienced mental health staff performed the data collection, both the index interview and the follow-up examination of medical records. The quality and quantity of information recorded during the follow-up period is very good since the Swedish personal identity number allows multiple service providers to share the same electronic medical record system, and makes feasible automatic updates from the population register. Age range, gender distribution and repetition rate in the present study population are similar to figures reported from previous studies on adult hospital-presenting self-harm patients [9, 41], which implies that our results might be applicable to similar clinical populations in other countries. The majority of the participants had inpatient treatment in connection with the index episode, which limits generalizability to hospital-treated self-harm patients. To the best of our knowledge, this is the first prediction study of the C-SSRS in such a population.

### Limitations

The number of suicides was not sufficient to analyse suicide as a separate outcome. Some participants may have made suicide attempts during the follow-up period without it coming to the attention of psychiatric services, and in this study, we only have knowledge of those episodes of self-harm that were mentioned in the medical records. Still, this is likely to give a more correct estimate of outcome events than only using data from the national diagnostic registers, since not all self-harm events are registered. While we do know that all patients without follow-up data were still alive six months after the index episode, we lack information on non-fatal suicide attempts for this small group.

## Conclusions

In this study of psychiatric patients with self-harm, suicidal ideation intensity and the C-SSRS total score were associated with increasing odds for non-fatal and fatal suicide attempt during a six-month follow-up. The associations, however, are not specific enough to guide treatment. Since the C-SSRS is widely used internationally, further studies could investigate its predictive properties in varied settings and cultures. With the exception of one item, the interrater reliability estimates indicate that the instrument works well for classification, for which it was originally developed.

## Abbreviations

AUC: Area under the curve
CI: Confidence interval
C-SSRS: Columbia-Suicide Severity Rating Scale
NSSI: Non-suicidal self-injury
OR: Odds ratio
PABAK: Prevalence-adjusted, bias-adjusted kappa
ROC: Receiver operating characteristic.
SA: Suicide attempt
SD: Standard deviation
SI: Suicidal ideation
